# 

**Published:** 1877-09

**Authors:** 


### ANNOUNCEMENTS FOR THE MONTH.

Societies.—Mondays, September 3d and 17th, Chicago Medical Society. Mondays, September 10th and 24th, Chicago Society of Physicians and Surgeons.

Clinics.—Saturdays, at Rush College, at 2 p. m. Surgical, Prof. Gunn. Tuesdays, at Chicago Medical College, 9 a. m. Surgical, Prof. Hyde.

Lectures.—Mondays, 4 p. m., at Cook County Hospital Necroscopy Theatre. On Pathological Anatomy, Prof. I. N. Danforth.

